# The Combination of Fat Distribution and BMI Redefines Obesity: Result From NHANES

**DOI:** 10.1002/jcsm.70013

**Published:** 2025-07-15

**Authors:** Liuqing Yang, Yizhong Ge, Qiankun Zhu, Qi Zhang, Lin Wang, Xin Wang, Yun Yang, Hanping Shi

**Affiliations:** ^1^ Department of Clinical Nutrition Beijing Shijitan Hospital, Capital Medical University Beijing China; ^2^ Key Laboratory of Cancer FSMP for State Market Regulation Beijing China; ^3^ Laboratory for Clinical Medicine Capital Medical University Beijing China; ^4^ The Second Affiliated Hospital and Yuying Children's Hospital of Wenzhou Medical University Wenzhou China; ^5^ Department of Orthopedics Surgery Beijing Chest Hospital, Capital Medical University, Beijing Tuberculosis and Thoracic Tumor Research Institute Beijing China; ^6^ Department of Genetics Yale School of Medicine New Haven Connecticut USA; ^7^ Department of General Surgery/Center for Cancer Nutrition and Metabolism Beijing Shijitan Hospital, Capital Medical University Beijing China; ^8^ National Clinical Research Center for Geriatric Diseases Xuanwu Hospital, Capital Medical University Beijing China

**Keywords:** BMI, centripetal obesity, obesity

## Abstract

**Background:**

Body mass index (BMI) has well‐recognized limitations, particularly in the context of the ‘obesity paradox’, where higher BMI does not consistently evaluate adverse outcomes. These limitations underscore the need for alternative approaches. This study aimed to redefine obesity using anthropometric indicators and to assess their prognostic value for mortality.

**Methods:**

The original cohort included participants from the US National Health and Nutrition Examination Survey (NHANES) from 1999 to 2006. Additional dual‐energy X‐ray absorptiometry (DXA) data on fat distribution were sourced from NHANES 1999–2006 and 2011–2018. Ten fat distribution indicators were adopted to assess the fat distribution in the target population, including five centripetal obesity indicators, three global obesity indicators and two limb obesity indicators. All‐cause, cardiovascular‐specific and cancer‐specific mortality outcomes were examined using weighted Cox proportional hazards models. Logistic regression was employed to evaluate associations between fat distribution indicators and the prevalence of specific diseases.

**Results:**

This study included 14 936 participants (weighted 152 823 236 participants, mean age: 45.56 ± 17.60 years) and two DXA‐assessed cohorts. Fat distribution indicators were correlated with various DXA‐defined fat components. All 10 indicators demonstrated significant associations with mortality. Notably, the relationship between centripetal obesity indicators and all‐cause mortality was approximately linear in both sexes. Centripetal obesity was also strongly associated with cardiovascular and cancer‐specific mortality (*p* < 0.001). Although fat distribution indicators were significantly linked to the prevalence of cardiovascular disease (CVD), they showed no clear association with cancer incidence. Individuals with obesity but without centripetal fat accumulation exhibited a similar or slightly higher mortality risk compared with those with normal BMI and no centripetal obesity.

**Conclusion:**

Centripetal obesity indicators emerged as the strongest independent evaluators of mortality, regardless of BMI classification. These findings highlight the clinical value of incorporating fat distribution metrics alongside BMI to more accurately assess obesity‐related mortality risk.

## 1 Introduction

Although obesity is conventionally diagnosed using body mass index (BMI), it is increasingly recognized that BMI alone is inadequate for clinical assessment, given its well‐documented limitations in capturing individual‐level adiposity‐related health risks [1]. The World Health Organization (WHO) redefines obesity as ‘abnormal or excessive fat accumulation that may impair health’ [1]. Over the past few decades, numerous studies have demonstrated that BMI may not accurately capture obesity in specific populations, such as patients with cardiovascular disease or cancer. In these groups, BMI‐defined obesity has paradoxically been associated with lower mortality and more favourable treatment outcomes [2, 3, 4, 5].

Recently, the European Association for the Study of Obesity published a new framework of the obesity stage in adults [6]. This framework is based on two components: an anthropometric component and a clinical component. BMI is no longer considered sufficient on its own, as it fails to capture critical variations in body fat distribution. Even within the anthropometric component, diagnosis requires both BMI and measures of fat distribution, such as the waist‐to‐height ratio (WHtR) [7].

In 1996, the WHO published a report that re‐evaluated the application of anthropometric measures across different age groups for assessing health, nutritional status, and social well‐being. BMI was found to be unsuitable for use in all individuals. They also recommended to use other anthropometric components for defining obesity [8]. In recent years, many studies had reported the association between various anthropometric indicators and survival. In the Framingham Study, the researchers identified BMI‐based obesity and centripetal obesity—measured by the subscapular‐to‐triceps skinfold ratio (STR)—as independent risk factors for cardiovascular disease (CVD) [9]. In the Honolulu Heart Program, subscapular skinfold (SSF) was shown to be an independent evaluator of stroke, whereas BMI was not associated with central fat accumulation [10]. Moreover, male patients with centripetal obesity were shown to have an increased risk of coronary heart disease, independent of BMI [11]. Similarly, in the participants with cancer, researchers used centripetal obesity measures—waist circumference (WC), WHtR and waist‐to‐hip ratio (WHR)—to compare with BMI‐based obesity classifications. The comparison revealed that WC [12], WHtR [13] and WHR [14] were more strongly associated with mortality in cancer patients than BMI. These findings suggest that BMI‐defined obesity differs substantially from obesity defined by centripetal fat distribution indicators [15]. Indicators such as WHR and STR may more effectively reflect fat distribution than BMI [16]. However, systematic investigations into anthropometry‐based fat distribution indices remain limited.

Building on our previous research and motivated by the need to refine obesity definitions, the present study utilizes anthropometric indicators to assess obesity [17] and evaluates the clinical relevance (CVD and cancer) and prognostic value of the proposed criteria in evaluating disease‐specific survival.

## 2 Methods

### 2.1 Study Population

The National Health and Nutrition Examination Survey (NHANES), administered by the US National Center for Health Statistics, has conducted biennial survey cycles since 1999 to monitor the health and nutritional status of the US population. All NHANES protocols received approval from the National Center for Health Statistics Ethics Review Board, and written informed consent was obtained from all participants.

The base participants were from four NHANES cycles conducted between 1999 and 2006. To examine the relationship between anthropometric indices and dual‐energy X‐ray absorptiometry (DXA) measurements, we extracted relevant DXA data for correlation analysis. DXA measurements were available in two parts in the NHANES database: 1999–2006 and 2011–2018. The 1999–2006 DXA dataset exhibited substantial missing data due to technical limitations during data collection. To address this issue, NHANES administrators applied multiple imputation procedures following predefined protocols (see https://wwwn.cdc.gov/Nchs/Nhanes/Dxa/Dxa.aspx). In contrast, the 2011–2018 DXA cohort was restricted to participants aged < 65 years, reflecting updated eligibility criteria for body composition assessment. DXA fat factors included trunk fat, total fat, left arm fat, right arm fat, left leg fat and right leg fat.

Mortality data were obtained from the NHANES Public Use Linked Mortality Files. These files provide mortality follow‐up information via linkage to the National Death Index through December 31, 2019. The underlying cause of death was coded using the UCOD LEADING variable and classified as all‐cause death [18]. The median follow‐up duration was 204.9 months for the 1999–2006 cohort and 59.2 months for the 2011–2019 cohort.

The inclusion criteria for this study were as follows: (1) age ≥ 18 years; (2) having measured triceps skinfold (TSF), Ac, BMI, WC, maximal calf circumference (CC), SSF and thigh circumference (TC); and (3) having neutrophil and lymphocyte ratio (NLR) (Figure S1).

### 2.2 Patient Characteristics

For the NHANES, information on cancer and CVDs, including diagnoses and disease types, was collected through in‐person interviews. Participants were classified as having cancer if they answered ‘yes’ to the following question: ‘Have you ever been told by a doctor or other health professional that you had cancer or a malignancy of any kind?’ Individuals who responded ‘yes’ were defined as cancer survivors and were then asked, ‘What kind of cancer was it?’. Participants were recorded as having CVDs if they answered ‘yes’ to the following question: ‘Has a doctor or other health professional ever told you that you had congestive heart failure/coronary heart disease/angina pectoris/stroke?’. The BMI was recorded conventionally as weight (kg)/height^2^ (m^2^) and was categorized as low (< 20 kg/m^2^), normal (20–25 kg/m^2^ for the US), overweight (25–30 kg/m^2^) and obese (≥ 30 kg/m^2^). The NLR is measured as the neutrophil count (×10^9^)/lymphocyte count (×10^9^). Systemic inflammation was defined as an NLR > 3 [19].

### 2.3 Making Composite Indicators and Obesity Based on Fat Distribution Define

Prior studies have demonstrated that TSF and WHR are strongly associated with nutritional status and mortality [20, 21]. Ten fat distribution indicators based on TSF, WC and other six factors have been made in order to define the fat distribution:

Centripetal obesity indicators: (1) subscapular skinfold‐to‐waist circumference ratio (SWR) = subscapular skinfold (SSF)/WC, (2) arm circumference‐to‐waist circumference ratio (AWR) = arm circumference (Ac)/WC, (3) maximal calf circumference‐to‐waist circumference ratio (CWR) = CC/WC, (4) triceps skinfold‐to‐waist circumference ratio (TSFWR) = TSF/WC and (5) thigh circumference‐to‐waist circumference ratio (TCWR) = TC/WC.

Global obesity indicators: (6) weight‐to‐waist circumference ratio (WWR) = Weight/WC, (7) WHtR = Waist/Height and (8) subscapular‐to‐triceps skinfold thickness ratio (STR) = subscapular skinfold (SSF)/TSF.

Limb obesity indicators: (9) triceps skinfold‐to‐maximal calf circumference ratio (TSFCCR) = TSF/CC and (10) triceps skinfold‐to‐thigh circumference ratio (TSFTCR) = TSF/TC.

DXA fat distribution indicators: arm fat‐to‐trunk fat ratio (ATrR) = (left arm fat + right arm fat)/trunk fat, leg fat‐to‐trunk fat ratio (LTrR) = (left leg fat + right leg fat)/trunk fat, arm fat‐to‐total fat ratio (ATR) = (left arm fat + right arm fat)/total fat and leg fat‐to‐total fat ratio (LTR) = (left leg fat + right leg fat)/total fat.

### 2.4 Statistical Analyses

The analysis proceeded in three sequential phases: (1) correlation assessment of anthropometric indicators, (2) survival evaluation modelling and (3) validation against DXA‐derived body composition indicators. All statistical analyses were conducted with weighting to account for the NHANES survey design. The R software (version 4.0.2, http://www.rproject.org) was used for the data analysis. Continuous variables were presented as mean (SD), and categorical variables were presented as frequency (weighted percentage). Between‐sex comparisons were conducted using the chi‐square test for categorical variables and the Student's *t*‐test for continuous variables. The correlation was analysed by the *corrplot* package. Five‐knot‐restricted cubic splines (RCS) were used in the *rms* package and *ggplot2* package. Based on the RCS, 10 FDIs have been divided into three groups. FDIs with a risk of death decrease monotonically chose the cut‐off value at the beginning of the curve with the most dramatic change. Indicators with a U‐shaped curve defined the normal group in the low‐risk group in the RCS. WHtR and STR have the clinical cut‐off value. The cut‐off value of TSF was defined by *survminer* package basing on survival. All cut‐off values have been considered the difference between the male and female. The hazard ratios (HR) and the 95% confidence intervals (CI) of the participants were estimated by modelling the risk factors using multivariate Cox regression models, weighted by the *survey* package and for the other survival analysis by the *survival* package. Associations between disease prevalence and obesity indicators were examined using generalized linear regression models. Models were adjusted for age, ethnicity and NLR.

## 3 Results

### 3.1 Study Population

This study included 14 936 participants (weighted 152 823 236 participants) from NHANES 1999–2006. The mean age of participants was 45.56 ± 17.60 years. Male participants have high weight (male 80.49 kg vs. female 67.98 kg, *p* < 0.001), high Ac (male 32.48 cm vs. female 30.03 cm, *p* < 0.001), high TC (male 51.84 cm vs. female 50.83 cm, *p* < 0.001), high CC (male 37.92 cm vs. female 36.72 cm, *p* < 0.001), high WC (male 95.95 cm vs. female 89.63 cm, *p* < 0.001), but low TSF (male 13.12 mm vs. female 22.32 mm, *p* < 0.001) and low SSF (male 18.28 mm vs. female 20.17 mm, *p* < 0.001). The CVD prevalence is similar in male and female (weighted%: male 8.1% vs. female 7.5%, *p* = 0.333), but the female has a significantly higher cancer prevalence than male (weighted%: male 6.6% vs. female 9.5%, *p* < 0.001). Among 10 fat distribution indicators, only WHtR has no difference between male and female Table S1.

DXA measurements in NHANES were grouped into two cohorts: 1999–2006 and 2011–2018. The demographic characteristics of these cohorts are summarized in Tables S2 and S3.

### 3.2 Correlation Between Anthropometric Factors and Fat Distribution Indicators and DXA Factors

**FIGURE 1:**
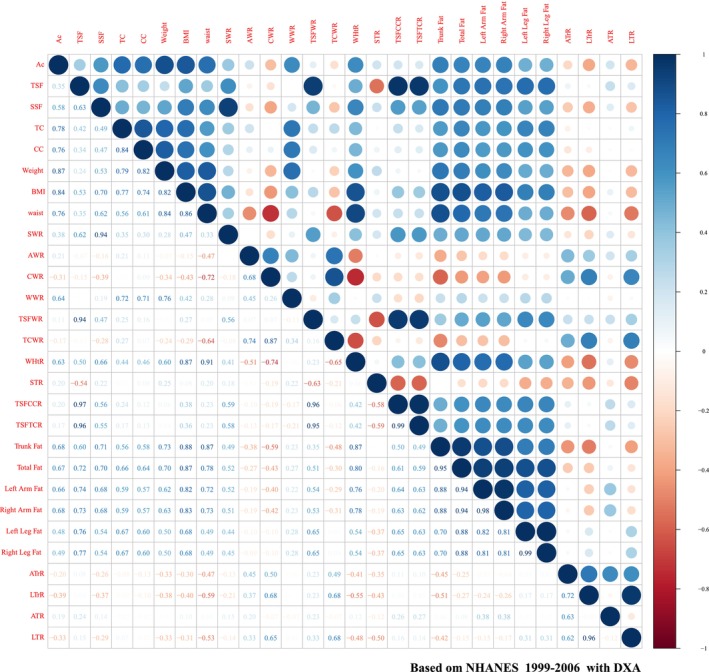
Correlation between factors and indicators, NHANES 1999–2006. ATrR: arm fat‐to‐trunk fat ratio; AWR: arm circumference‐to‐waist circumference ratio; CWR: maximal calf circumference‐to‐waist circumference ratio; LTrR: leg fat‐to‐trunk fat ratio; STR: subscapular‐to‐triceps skinfold thickness ratio; SWR: subscapular skinfold‐to‐waist circumference ratio; TCWR: thigh circumference‐to‐waist circumference ratio; TSFCCR: triceps skinfold‐to‐maximal calf circumference ratio; TSFTCR: triceps skinfold‐to‐thigh circumference ratio; TSFWR: triceps skinfold‐to‐waist circumference ratio; WHtR: waist circumference‐to‐height ratio; WWR: weight‐to‐waist circumference ratio. ATR = arm fat‐to‐total fat ratio; LTR = leg fat‐to‐total fat ratio.

Correlation analysis revealed that seven factors excluding TSF had moderate to strong correlation (*r* > 0.4). SSF also demonstrated a high correlation with the other six factors (*r* > 0.4). Very strong correlations were observed between fat factors and their corresponding centripetal obesity indicators (*r* > 0.8). TSF was moderately correlated with BMI (*r* = 0.54) and strongly correlated with SSF (*r* = 0.63). TSF has a very strong correlation (*r* > 0.8) with its associated indicators, including TSFWR, TSFCCR and TSFTCR. SSF also has a very strong correlation with SWR (*r* > 0.8). WHtR was highly correlated with all factors, especially a very strong correlation with BMI (Figure 1).

The anthropometric factors showed a moderate to strong correlation with DXA fat factors. The fat distribution indicators showed a different correlation with DXA fat factors or DXA fat distribution indicators. SWR, TSFWR, CWR and WHtR showed a high correlation with DXA fat factors, and CWR, TCWR STR showed a high correlation with DXA fat distribution indicators (Figure 1).

### 3.3 Defined the Obesity Based on 10 Fat Distribution Indicators (Cut‐Off Value)

**FIGURE 2:**
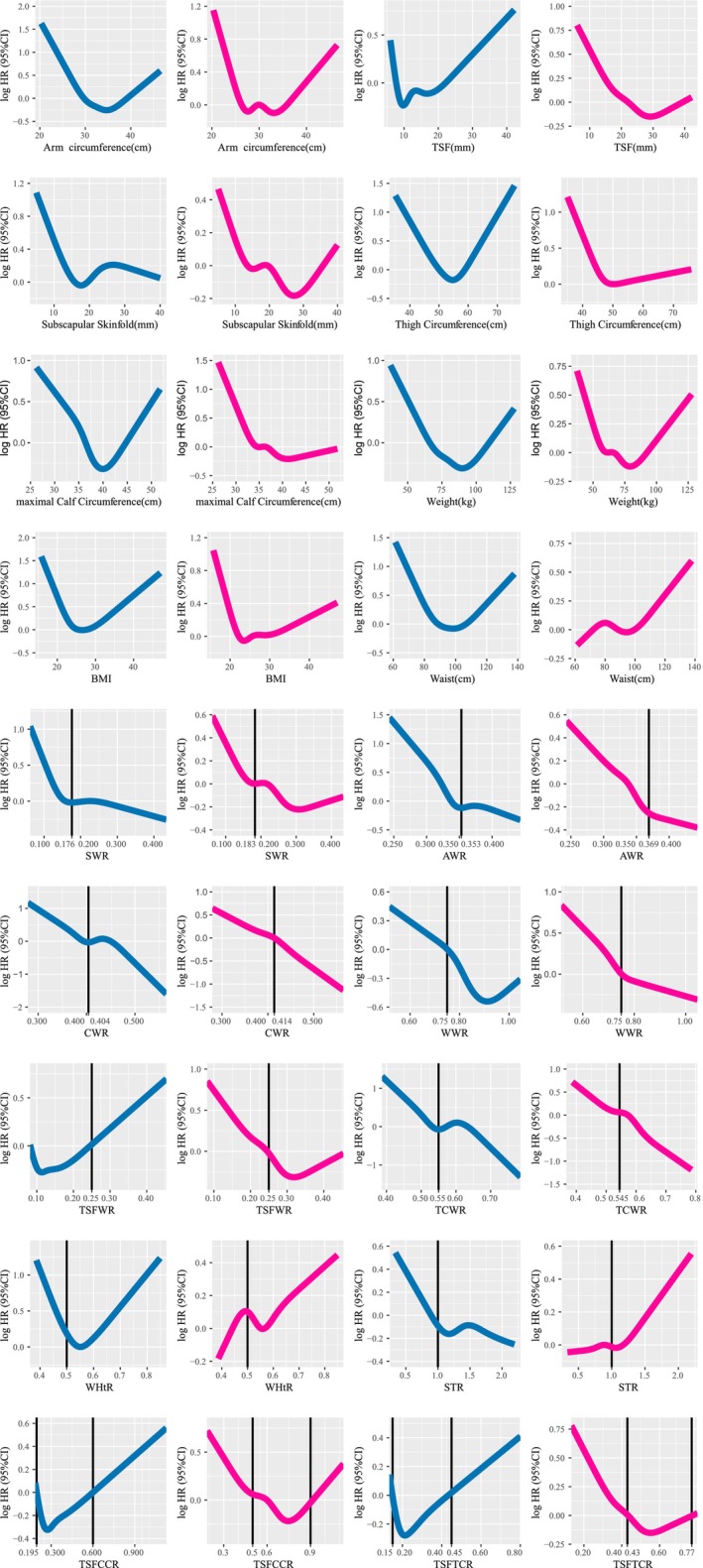
Restricted spline curves (weighted) examining the association of 16 indicators and all‐cause mortality, NHANES 1999–2006. Weighted to be nationally representative, values are expressed as *n* (weighted %). Adjusted by age, ethnicity and NLR. AWR: arm circumference‐to‐waist circumference ratio; CWR: maximal calf circumference‐to‐waist circumference ratio; STR: subscapular‐to‐triceps skinfold thickness ratio; SWR: subscapular skinfold‐to‐waist circumference ratio; TCWR: thigh circumference‐to‐waist circumference ratio; TSFCCR: triceps skinfold‐to‐maximal calf circumference ratio; TSFTCR: triceps skinfold‐to‐thigh circumference ratio; TSFWR: triceps skinfold‐to‐waist circumference ratio; WHtR: waist circumference‐to‐height ratio; WWR: weight‐to‐waist circumference ratio.

RCS analysis revealed that sex significantly influenced eight anthropometric factors, but the 10 fat distribution indicators were not strongly affected by the sex. Consistent with Table S1, RCS results indicated that, within the protective range, males had higher weight, AC, TC and CC but lower TSF and SSF than females. The eight anthropometric factors exhibited U‐shaped associations with mortality, with notable differences in distribution between males and females. The 10 fat distribution indicators were categorized into three different groups. Centripetal obesity indicators displayed a linear association with mortality in RCS analysis. In contrast, global obesity indicators and limb obesity indicators had U‐shaped or J‐shaped curves with mortality. The distribution of centripetal obesity indicators was consistent across sexes, whereas global obesity indicators and limb obesity indicators varied significantly by sexes. Cut‐off values for 10 fat distribution indicators were determined based on their respective RCS curve (Figure 2). These cut‐off values are listed in Table S4.

### 3.4 Survival Evaluation Ability of 10 Fat Distribution Indicators

When analysed as continue indicators, 10 fat distribution indicators were significantly association with all‐cause mortality (*p* < 0.001) (Figure 1 and Table S5). Participants with low centripetal obesity indicators had significantly higher all‐cause mortality across all subgroups: the total participants (Table 1), participants with CVD, participant with cancer, participants without cancer or CVD, elderly participants (≥ 65 years) and young participants (< 65 years) (Table S6) (all *p* < 0.001). Low centripetal obesity indicators were also associated with increased risk of CVD‐specific mortality (Table 2) and cancer‐specific mortality (Table 3). TSFWR was the best discriminating indicator among the five centripetal obesity indicators.

**TABLE 1:** The association of 10 indicators (cut‐off) and all‐cause mortality, NHANES 1999–2006, adjusted by age, ethnicity and NLR.

	Crude HR (95% CI)	*p*	Adjusted HR (95% CI)	*p*
SWR				
High	Ref.		Ref.	
Low	1.349 (1.257, 1.448)	*p* < 0.05	1.282 (1.180, 1.392)	*p* < 0.05
AWR				
High	Ref.		Ref.	
Low	3.850 (3.487, 4.251)	*p* < 0.05	1.479 (1.335, 1.639)	*p* < 0.05
CWR				
High	Ref.		Ref.	
Low	4.085 (3.690, 4.522)	*p* < 0.05	**1.524 (1.373, 1.692)**	*p* < 0.05
WWR				
High				
Low	2.501 (2.290, 2.731)	*p* < 0.05	1.137 (1.038, 1.246)	*p* < 0.05
TSFWR				
High	Ref.		Ref.	
Low	1.685 (1.481, 1.917)	*p* < 0.05	**1.587 (1.430, 1.762)**	*p* < 0.05
TCWR				
High	Ref.		Ref.	
Low	5.064 (4.587, 5.592)	*p* < 0.05	1.517 (1.382, 1.665)	*p* < 0.05
WHtR				
High	Ref.		Ref.	
Low	0.406 (0.358, 0.46)	*p* < 0.05	1.034 (0.929, 1.151)	**0.544**
STR				
High	Ref.		Ref.	
Low	1.044 (0.963, 1.131)	0.300	0.861 (0.796, 0.932)	*p* < 0.05
TSFCCR				
Low	1.032 (0.908, 1.173)	0.628	1.253 (1.129, 1.390)	*p* < 0.05
Normal	Ref.		Ref.	
High	1.360 (1.088, 1.699)	*p* < 0.05	1.440 (1.158, 1.790)	*p* < 0.05
TSFTCR				
Low	0.948 (0.861, 1.043)	0.275	1.117 (1.032, 1.209)	*p* < 0.05
Normal	Ref.		Ref.	
High	1.654 (1.237, 2.211)	*p* < 0.05	1.521 (1.154, 2.006)	*p* < 0.05
BMI				
Low	1.034 (0.869, 1.229)	0.709	1.676 (1.409, 1.993)	*p* < 0.05
Normal	Ref.		Ref.	
High	1.289 (1.138, 1.459)	*p* < 0.05	1.122 (1.002, 1.255)	*p* < 0.05
TSF				
High	Ref.		Ref.	
Low	1.055 (0.958, 1.161)	0.276	1.162 (1.074, 1.258)	*p* < 0.05

*Note:* The cut‐off value is shown in Table S4.

Abbreviations: AWR: arm circumference‐to‐waist circumference ratio; CWR: maximal calf circumference‐to‐waist circumference ratio; STR: subscapular‐to‐triceps skinfold thickness ratio; SWR: subscapular skinfold‐to‐waist circumference ratio; TCWR: thigh circumference‐to‐waist circumference ratio; TSFCCR: triceps skinfold‐to‐maximal calf circumference ratio; TSFTCR: triceps skinfold‐to‐thigh circumference ratio; TSFWR: triceps skinfold‐to‐waist circumference ratio; WHtR: waist circumference‐to‐height ratio; WWR: weight‐to‐waist circumference ratio.

**TABLE 2:** The association of 10 indicators (cut‐off) and CVD‐specific mortality, NHANES 1999–2006, adjusted by age, ethnicity and NLR.

	Crude HR (95% CI)	*p*	Adjusted HR (95% CI)	*p*
SWR				
High	Ref.		Ref.	
Low	1.216 (1.043, 1.418)	*p* < 0.05	1.102 (0.937, 1.296)	0.242
AWR				
High	Ref.		Ref.	
Low	4.766 (3.662, 6.201)	*p* < 0.05	1.624 (1.201, 2.196)	*p* < 0.05
CWR				
High	Ref.		Ref.	
Low	5.018 (4.287, 5.872)	*p* < 0.05	1.691 (1.456, 1.963)	*p* < 0.05
WWR				
High	Ref.		Ref.	
Low	2.86 (2.469, 3.313)	*p* < 0.05	1.154 (1.008, 1.321)	*p* < 0.05
TSFWR				
High	Ref.		Ref.	
Low	2.042 (1.604, 2.599)	*p* < 0.05	**1.884 (1.52, 2.335)**	*p* < 0.05
TCWR				
High	Ref.		Ref.	
Low	6.875 (5.437, 8.694)	*p* < 0.05	**1.851 (1.466, 2.337)**	*p* < 0.05
WHtR				
High	Ref.		Ref.	
Low	0.282 (0.211, 0.377)	*p* < 0.05	0.786 (0.611, 1.011)	**0.061**
STR				
High	Ref.		Ref.	
Low	0.964 (0.835, 1.114)	0.621	0.763 (0.653, 0.892)	*p* < 0.05
TSFCCR				
Low	1.059 (0.844, 1.33)	0.620	1.253 (1.011, 1.553)	*p* < 0.05
Normal	Ref.		Ref.	
High	1.255 (0.843, 1.869)	0.264	1.350 (0.917, 1.987)	0.128
TSFTCR				
Low	0.89 (0.732, 1.083)	0.244	1.018 (0.837, 1.240)	0.855
Normal	Ref.		Ref.	
High	1.538 (0.926, 2.555)	0.096	1.405 (0.877, 2.252)	0.158
BMI				
Low	1.188 (0.858, 1.646)	0.300	1.977 (1.405, 2.783)	*p* < 0.05
Normal	Ref.		Ref.	
High	1.614 (1.315, 1.981)	*p* < 0.05	1.415 (1.156, 1.731)	*p* < 0.05
TSF				
High	Ref.		Ref.	
Low	1.049 (0.887, 1.24)	0.579	1.119 (0.963, 1.301)	0.141

*Note:* The cut‐off value is shown in Table S4.

Abbreviations: AWR: arm circumference‐to‐waist circumference ratio; CWR: maximal calf circumference‐to‐waist circumference ratio; STR: subscapular‐to‐triceps skinfold thickness ratio; SWR: subscapular skinfold‐to‐waist circumference ratio; TCWR: thigh circumference‐to‐waist circumference ratio; TSFCCR: triceps skinfold‐to‐maximal calf circumference ratio; TSFTCR: triceps skinfold‐to‐thigh circumference ratio; TSFWR: triceps skinfold‐to‐waist circumference ratio; WHtR: waist circumference‐to‐height ratio; WWR: weight‐to‐waist circumference ratio.

**TABLE 3:** The association of 10 indicators (cut‐off) and cancer‐specific mortality, NHANES 1999–2006, adjusted by age, ethnicity and NLR.

	Crude HR (95% CI)	*p*	Adjusted HR (95% CI)	*p*
SWR				
High	Ref.		Ref.	
Low	1.233 (1.040, 1.462)	*p* < 0.05	1.217 (1.017, 1.455)	*p* < 0.05
AWR				
High	Ref.		Ref.	
Low	3.523 (2.814, 4.411)	*p* < 0.05	1.600 (1.276, 2.007)	*p* < 0.05
CWR				
High	Ref.		Ref.	
Low	3.909 (3.184, 4.799)	*p* < 0.05	**1.704 (1.366, 2.125)**	*p* < 0.05
WWR				
High	Ref.		Ref.	
Low	1.568 (1.284, 1.915)	*p* < 0.05	0.786 (0.635, 0.973)	*p* < 0.05
TSFWR				
High	Ref.		Ref.	
Low	1.590 (1.281, 1.974)	*p* < 0.05	1.535 (1.218, 1.934)	*p* < 0.05
TCWR				
High	Ref.		Ref.	
Low	4.818 (3.957, 5.868)	*p* < 0.05	**1.785 (1.459, 2.184)**	*p* < 0.05
WHtR				
High	Ref.		Ref.	
Low	0.456 (0.355, 0.584)	*p* < 0.05	1.011 (0.785, 1.302)	**0.931**
STR				
High	Ref.		Ref.	
Low	0.881 (0.731, 1.061)	0.182	0.755 (0.635, 0.898)	*p* < 0.05
TSFCCR				
Low	0.813 (0.662, 0.999)	*p* < 0.05	0.982 (0.799, 1.206)	0.862
Normal	Ref.		Ref.	
High	1.562 (1.072, 2.278)	*p* < 0.05	1.621 (1.093, 2.405)	*p* < 0.05
TSFTCR				
Low	0.793 (0.670, 0.939)	*p* < 0.05	0.931 (0.789, 1.099)	0.399
Normal	Ref.		Ref.	
High	1.864 (1.110, 3.130)	*p* < 0.05	1.728 (1.029, 2.902)	*p* < 0.05
BMI				
Low	0.694 (0.476, 1.012)	0.058	1.070 (0.701, 1.633)	0.755
Normal	Ref.		Ref.	
High	1.168 (0.919, 1.483)	0.204	**1.011 (0.808, 1.267)**	**0.921**
TSF				
High	Ref.		Ref.	
Low	0.915 (0.773, 1.084)	0.306	1.023 (0.859, 1.218)	0.802

*Note:* The cut‐off value is shown in Table S4.

Abbreviations: AWR: arm circumference‐to‐waist circumference ratio; CWR: maximal calf circumference‐to‐waist circumference ratio; STR: subscapular‐to‐triceps skinfold thickness ratio; SWR: subscapular skinfold‐to‐waist circumference ratio; TCWR: thigh circumference‐to‐waist circumference ratio; TSFCCR: triceps skinfold‐to‐maximal calf circumference ratio; TSFTCR: triceps skinfold‐to‐thigh circumference ratio; TSFWR: triceps skinfold‐to‐waist circumference ratio; WHtR: waist circumference‐to‐height ratio; WWR: weight‐to‐waist circumference ratio.

Participants with low BMI had elevated risks of all‐cause mortality and CVD‐associated mortality (Tables 1 and 2). However, obesity (high BMI) was not significantly associated with all‐cause mortality in participants with CVD [HR (95% CI): 1.043 (0.883, 1.232), *p* = 0.624], participants with cancer [HR (95% CI): 0.940 (0.772, 1.146), *p* = 0.542], participants without cancer or CVD [HR (95% CI): 1.170 (1100.368), *p* = 0.050] and elderly participants (≥ 65 years) [HR (95% CI): 1.059 (0.944, 1.188), *p* = 0.326] (Table S6). Participants classified as obese based on centripetal fat indicators had a higher mortality risk than those classified by BMI alone.

Limb fat indicators showed inconsistent evaluative value for mortality. Obesity defined by WHtR was not associated with increased mortality risk in all participants and subgroup analysis (Tables 1, 2, 3 and S4–S8). Similarly, STR and WWR (Table S6) were not significantly associated with mortality in certain subgroups (*p* > 0.05). Notably, low WWR demonstrated a protective association with cancer‐associated mortality [HR (95% CI): 0.786 (0.635, 0.973), *p* = 0.027] (Table 3).

### 3.5 Association Between the Prevalence of Special Diseases and Indicators

All 10 fat distribution indicators were significantly associated with the prevalence of CVD (*p* < 0.05), whereas only TSFWR and WWR were associated with cancer prevalence (Table S7). BMI showed a similar pattern: High BMI was significantly associated with CVD prevalence (*p* < 0.05), but not with cancer prevalence.

### 3.6 The Relationship Between Fat Distribution and Obesity

Low BMI was associated with increased mortality risk. Participants with low TSFWR predominantly overlapped with those participants with low BMI. Among the five centripetal obesity indicators, TSFWR identified the largest number of participants with centripetal obesity, whereas WWR identified the fewest (Table S8).

Survival analysis revealed that the low BMI with centripetal obesity had the highest risk of all‐cause mortality. Obesity participants exhibited a slightly higher or comparable risk of all‐cause mortality compared with those with normal BMI and no centripetal obesity. For example, participants with low AWR and normal BMI had significantly increased mortality risk [low AWR + normal BMI: HR (95% CI): 1.517 (1.3361723), *p* < 0.001; high AWR + high BMI: HR (95% CI): 1.293 (1.011, 1.655), *p* = 0.041]. Analysis based on centripetal obesity fat distribution indicators further showed that obese participants without centripetal obesity have a lower risk of all‐cause mortality compared with those with normal BMI but with centripetal obesity. In this context, the absence of centripetal obesity appeared to have a protective effect among individuals with high BMI (Figure S2).

### 3.7 The Association Between DXA Fat Distribution Indicator LTrR and Survival

In both the NHANES 1999–2006 and 2011–2018 cohorts, the leg‐to‐trunk fat ratio (LTrR) exhibited a linear inverse association with all‐cause mortality (Figure S3). Higher LTrR was associated with improved survival: [NHANES 1999–2006: HR = 0.817; 95% CI: 0.713–0.936; *p* < 0.05] and [NHANES 2011–2018: HR = 0.468; 95% CI: 0.267–0.820; *p* < 0.05] (Table S9).

## 4 Discussion

Anthropometry factors are simple and efficient factors in nutritional assessment [22]. BMI has long been used as a standard measure to define obesity and guide health management. However, numerous studies have found the obesity paradigm based on the BMI [17]. Our findings demonstrated a J‐ or U‐shaped relationship between traditional anthropometric factors (such as BMI) and mortality, whereas centripetal obesity indicators showed a consistent linear association with mortality risk. Correlation analysis showed that WHtR was very strongly correlated with both BMI and DXA‐derived fat measurements, whereas indicators based on TSF were primarily associated with TSF and SSF alone. Survival analysis showed the five centripetal obesity indicators were strongly associated with the mortality, whereas other indicators lacked consistent evaluative power. Combined analysis of BMI and fat distribution demonstrated that centripetal obesity indicators showed an independent association with mortality. All indicators were significantly associated with CVD prevalence, though few showed associations with cancer prevalence. Obesity defined by the DXA‐based LTrR indicator was associated with reduced mortality, suggesting that fat distribution‐based definitions may better capture individuals at highest health risk.

Our previous studies have demonstrated that the widely used BMI‐based definition of obesity remains highly controversial [17]. This controversy is often referred to as the ‘obesity paradox’. It describes the phenomenon in which obesity, as measured by BMI, does not consistently correlate with reduced survival time. This paradox is particularly evident in patients with CVD and cancer. Anthropometric measures, including BMI, are widely used because they are simple, accessible and relatively objective. In light of our prior findings and the growing need to refine the definition of obesity, this study focuses on evaluating obesity using anthropometry‐based indicators and investigates whether these refined definitions, which incorporate disease relevance, improve the evaluation of survival outcomes.

Anthropometry factors are strongly influenced by sex. Previous studies have shown that anthropometry factors exhibit sex‐specific associations such as waist [23] or TSF [24]. In our study, many anthropometric factors showed a different shape in male and female, whereas the cut‐off value should be defined based on different sexes. WHtR is a widely used index among many fat distribution indicators [11, 25]. In our research, WHtR was highly correlated with BMI. WHtR also exhibited a similar U‐shaped RCS curve with BMI. Further analyses showed that none of the three global obesity indicators outperformed BMI in evaluative survival. The need for different cut‐off values across sexes may hinder the general applicability of these factors. Notably, some fat distribution indicators showed consistent distributions and linear associations with mortality across both sexes. For each centripetal obesity indicator, we defined a unified cut‐point and found that low centripetal obesity indicators were consistently associated with a high risk of mortality across all subgroups. TSFWR demonstrated the strongest evaluative value for mortality among the centripetal indicators.

Previous studies have identified TSF as a reliable anthropometric factor for evaluating survival in patients with cancer [20]. TSF and SSF, as independent fat indices, more accurately reflect upper limb fat levels. In our study, TSF or SSF showed weak correlations with other anthropometric factors. Corresponding fat distribution indexes involving TSF and SSF also exhibited strong independence from other anthropometric factors. TSF has been considered a more direct indicator of body fat than BMI in studies using DXA as the reference [26]. However, an analysis by *Freedman DS* et al. using NHANES data concluded that the TSF may not be suitable for defining obesity [27]. That study also highlighted substantial sex‐based differences in TSF measurements [27]. Our findings similarly demonstrated significant sex‐related variation in TSF. However, when TSF or SSF was combined with WC to form centripetal obesity indicators, similar patterns of fat distribution were observed across sexes. These centripetal obesity indicators demonstrated good generalizability across genders. The composite nature of these indicators likely mitigates the differential effects of sex on body composition. Since sex‐based differences in body composition are distributed across multiple regions, combined indicators may more effectively neutralize their confounding effects on mortality evaluation. In other studies, it was also shown that the differences in the composite indicators between genders were smaller. Although there were differences in the calculated cut‐off values for metabolic syndrome, these differences were relatively small [28]. The reasons for the smaller gender differences shown by the composite indicators still require further research.

In survival analysis, centripetal obesity indicators demonstrated strong evaluative value, likely due to their linear association with mortality. Low centripetal obesity indicators were associated with an increased risk of death. These findings are consistent with prior studies involving the WHR. For example, Irfan Khan reported the superiority of the WHR based on the UK Biobank. Their findings showed a linear relationship between WHR and mortality, with a higher WHR value associated with greater mortality risk [21]. The protective effect of high BMI in individuals with chronic diseases, especially those with CVD or cancer or the elderly participants, has been discovered by many researchers and is called the ‘obesity paradigm’ [17, 29, 30].

Subgroup analyses in our study further confirmed the presence of the obesity paradox for BMI. High body weight alone did not appear to be a risk factor for poor survival. Centripetal obesity indicators capture fat distribution using combinations of waist circumference and limb fat measurements. Across subgroups, centripetal obesity was consistently associated with increased all‐cause mortality. In cause‐specific mortality analysis, high BMI was a protective factor against CVD death and cancer‐specific death, whereas centripetal obesity indicators were associated with increased risk. Similar findings from WHR‐based survival analyses further support the notion that fat distribution is a more accurate evaluator of mortality than BMI‐defined obesity [21]. The observed paradoxical protective effect of overweight BMI classifications may, in part, be explained by failure to account for centripetal fat distribution [31].

In previous analyses of NHANES, other researchers reported that the WHtR was positively associated with mortality risk, similar to BMI [25]. However, this research did not account for sex‐specific differences. In contrast, our study found that the global obesity indicators including WHtR differed significantly between male and female. For instance, a 1986 study found that centripetal obesity, as measured by the STR, was associated with the incidence of diabetes only in female [32]. In our research, however, centripetal obesity indicators had a similar association with mortality across both sexes. Therefore, we conducted our primary analyses in the overall participants rather than stratifying by sex.

Previous studies have shown that CC, TSF, AC and TC were associated with mortality and quality of life [33, 34]. As illustrated in Figure 2, these anthropometric factors exhibited a U‐shaped association with mortality. Similarly, limb obesity indicators also demonstrated U‐shaped associations with mortality. However, in the subgroup analysis, the limb obesity showed an unstable association with mortality. This inconsistency may be due to sex‐related differences in limb fat distribution. Limb obesity indicators varied substantially between males and females. In contrast, in our research, centripetal obesity indicators showed a stable and same association with mortality in different sexes. These indicators were more effective in identifying individuals at higher mortality risk.

Our study demonstrated that the evaluative value of the centripetal obesity indicators is independent of obesity defined by BMI. Normal‐BMI participants with centripetal obesity had higher all‐cause mortality than those obese participants without centripetal obesity. Consistent with our findings, the Honolulu Heart Program and Framingham studies reported that centripetal obesity was associated with an increased risk of CVD, independent of BMI [16, 18]. WHR‐defined centripetal obesity was also an independent risk factor for breast cancer among Indian women [19]. Centripetal obesity in middle age increased the risk of Alzheimer's disease in long‐term follow‐up, and the effect was independent of obesity defined by BMI [35]. These results suggest that BMI and centripetal obesity indicators are different indicators, and centripetal obesity is the most dangerous type of obesity [20]. In our study, centripetal obesity indicators were consistently associated with CVD prevalence, although only some were associated with cancer prevalence. Therefore, combining fat distribution indices with BMI may provide a more accurate assessment of an individual's obesity status and associated health risks.

Anthropometric indicators are based on external physical measurements and therefore may lack precision in assessing internal body composition. To address this, we compared anthropometric indicators with body composition metrics derived from DXA. Our findings showed that anthropometric indicators were moderately to strongly correlated with DXA‐based fat measurements. Moreover, DXA‐derived data more accurately identified individuals at higher risk of mortality. Thus, different indicators may be appropriately applied to assess fat distribution under varying clinical or population‐based conditions.

Our study has some limitations. First, although hip circumference was measured in NHANES, it was not consistently measured alongside other fat‐related anthropometric measures. As a result, WHR was not directly compared with centripetal obesity indicators in this analysis. Second, prior studies have shown that longitudinal BMI trajectory analysis provides stronger evaluative power for disease risk than cross‐sectional measurement [36]. Multiple measurements can provide a more accurate analysis for the judgement of obesity level. Only one measurement cannot describe the fat distribution of participants comprehensively, especially considering that TSF was always affected by activity. Third, although DXA is the most widely accepted method of measuring body composition due in part to its speed, ease of use and low radiation exposure (introduction from NHANES), the limitations related to the DXA dataset must also be considered. The NHANES 2011–2018 DXA sample was limited to participants under 65 years of age and had a relatively short follow‐up period. Although we aimed to further assess the potential of fat distribution indicators in refining obesity definitions using DXA, the available datasets may not be fully representative of the general population. In this study, DXA data were used selectively to validate the stability and objectivity of anthropometric indicators. Further research with repeated measures is warranted to better assess temporal changes in fat distribution and their clinical significance. Finally, our study focused primarily on all‐cause and disease‐specific mortality outcomes. Associations between fat distribution and disease prevalence were observational and cannot establish causality. BMI‐defined obesity has been linked to numerous chronic diseases [37]. Whether centripetal obesity confers similar or greater disease risk remains to be explored in future studies.

## 5 Conclusions

This study utilized anthropometric indicators to compare the association of centripetal obesity, global obesity and limb obesity distribution, as well as BMI‐defined obesity, with mortality and disease prevalence. Survival analysis demonstrated that all five centripetal obesity indicators were significantly associated with all‐cause mortality. Centripetal obesity remained an independent evaluator of mortality, irrespective of BMI classification. Anthropometric indicators showed moderate to strong correlations with DXA‐derived measures of body fat. Centripetal obesity indicators were also significantly associated with CVD prevalence. The combined use of fat distribution indices and BMI may offer a more comprehensive assessment of obesity‐related health risks. Further research is needed to validate the relationship between central obesity and disease onset.

## Conflicts of Interest

The authors declare no conflicts of interest.

## Supporting information


**Table S1** Sample size and characteristics, NHANES 1999–2006.
Table S2 Sample size and characteristics, NHANES 1999–2006 with DXA data.
Table S3 Sample size and characteristics, NHANES 2011–2018 with DXA data.
Table S4 Cut‐off value of 10 fat distribution indicators, NHANES 1999–2006, adjusted by age, ethnicity and NLR.
Table S5 The association of 10 indicators and all‐cause mortality, NHANES 1999–2006, adjusted by age, ethnicity and NLR.
Table S6 Sensitive analysis: The association of 10 indicators (cut‐off) and all‐cause mortality, NHANES 1999–2006, adjusted by age, ethnicity and NLR.
Table S7 Logistic regression analysis of the association between CVD prevalence and 10 indicators (cut‐off). NHANES 1999–2006, adjusted by age, ethnicity and NLR.
Table S8 Obesity (defined by BMI and 10 indicators) population distribution, NHANES 1999–2006.
Table S9 The association of LTrR and all‐cause mortality in all participants, adjusted by age, ethnicity and NLR.
Figure S1 Flow chat. *Note:* SWR: subscapular skinfold‐to‐waist circumference ratio; AWR: arm circumference‐to‐waist circumference ratio; CWR: maximal calf circumference‐to‐waist circumference ratio; TSFWR: triceps skinfold‐to‐waist circumference ratio; TCWR: thigh circumference‐to‐waist circumference ratio; WWR: weight‐to‐waist circumference ratio; WHtR: waist circumference‐to‐height ratio; STR: subscapular‐to‐triceps skinfold thickness ratio; TSFCCR: triceps skinfold‐to‐maximal calf circumference ratio; TSFTCR: triceps skinfold‐to‐thigh circumference ratio.
Figure S2 Association of the combination of fat distribution and BMI and all‐cause mortality, NHANES 1999–2006. Weighted to be nationally representative, values are expressed as *n* (weighted %). Adjusted by age, ethnicity and NLR. *Note:* SWR: subscapular skinfold‐to‐waist circumference ratio; AWR: arm circumference‐to‐waist circumference ratio; CWR: maximal calf circumference‐to‐waist circumference ratio; TSFWR: triceps skinfold‐to‐waist circumference ratio; TCWR: thigh circumference‐to‐waist circumference ratio; WWR: weight‐to‐waist circumference ratio; WHtR: waist circumference‐to‐height ratio; STR: subscapular‐to‐triceps skinfold thickness ratio; TSFCCR: triceps skinfold‐to‐maximal calf circumference ratio; TSFTCR: triceps skinfold‐to‐thigh circumference ratio.
Figure S3 Restricted spline curves (weighted) examining the association of LTrR and all‐cause mortality. Weighted to be nationally representative. Adjusted by age, ethnicity, NLR. Note: SWR: subscapular skinfold‐to‐waist circumference ratio; AWR: arm circumference‐to‐waist circumference ratio; CWR: maximal calf circumference‐to‐waist circumference ratio; TSFWR: triceps skinfold‐to‐waist circumference ratio; TCWR: thigh circumference‐to‐waist circumference ratio; WWR: weight‐to‐waist circumference ratio; WHtR: waist circumference‐to‐height ratio; STR: subscapular‐to‐triceps skinfold thickness ratio; TSFCCR: triceps skinfold‐to‐maximal calf circumference ratio; TSFTCR: triceps skinfold‐to‐thigh circumference ratio. LTrR = leg fat‐to‐trunk fat ratio.

## Data Availability

The data described in the manuscript, code book and analytic code will be made available upon request pending application and approval.
