# Supplementary material for: The Combination of Fat Distribution and BMI Redefines Obesity: Result From NHANES
Source: J Cachexia Sarcopenia Muscle. 2025 Jul 15;16(4):e70013. doi: 10.1002/jcsm.70013 (PMC12260474; doi:10.1002/jcsm.70013)

Fat distribution redefines obesity

Table S1 Sample size and characteristics, NHANES 1999 to 2006.

|  | **Total (14935)** | **Female (7381)** | **Male (7554)** | **P value** |
| --- | --- | --- | --- | --- |
| Sex n (weighted %) |  |  |  | p<0.05 |
| Female | 7381 (49.6) | 7381 (100.0) | 0 |  |
| Male | 7554 (50.4) | 0 | 7554 (100.0) | p<0.05 |
| Age, year mean (SD) | 45.56 (17.60) | 45.21 (18.15) | 45.90 (16.99) |  |
| Age n (weighted %) |  |  |  | p<0.05 |
| <45 year | 7814 (53.4) | 3954 (51.3) | 3860 (55.4) |  |
| 45-65 year | 3646 (30.4) | 1733 (30.1) | 1913 (30.6) |  |
| ≥65 year | 3475 (16.2) | 1694 (18.6) | 1781 (14.0) |  |
| BMI n (weighted %) |  |  |  | p<0.05 |
| <20 kg/m^2^ | 5259 (36.0) | 2708 (40.0) | 2551 (32.0) |  |
| 20-25 kg/m^2^ | 1089 (7.2) | 654 (9.7) | 435 (4.8) |  |
| 25-30 kg/m^2^ | 5694 (37.7) | 2475 (31.2) | 3219 (44.0) |  |
| ≥30 kg/m^2^ | 2893 (19.1) | 1544 (19.1) | 1349 (19.1) |  |
| Ethnic n (weighted %) |  |  |  | p<0.05 |
| Mexican American | 3520 (7.4) | 1725 (6.5) | 1795 (8.2) |  |
| Non-Hispanic Black | 2720 (8.9) | 1256 (8.8) | 1464 (9.0) |  |
| Non-Hispanic White | 7507 (73.7) | 3762 (74.2) | 3745 (73.3) |  |
| Other Hispanic | 616 (5.1) | 334 (5.4) | 282 (4.8) |  |
| Other Race - Including Multi-Racial | 572 (4.9) | 304 (5.2) | 268 (4.6) |  |
| CVD n (weighted %) |  |  |  | 0.333 |
| no | 11818 (92.2) | 5963 (92.5) | 5855 (91.9) |  |
| yes | 1353 (7.8) | 581 (7.5) | 772 (8.1) |  |
| Tumor n (weighted %) |  |  |  | p<0.05 |
| no | 12037 (92.0) | 5957 (90.5) | 6080 (93.4) |  |
| yes | 1122 (8.0) | 578 (9.5) | 544 (6.6) |  |
| Tumor type n (weighted %) | |  |  | p<0.05 |
| bladder | 22 (1.6) | 5 (0.5) | 17 (3.4) |  |
| blood | 2 (0.2) | 0 (0.0) | 2 (0.5) |  |
| bone | 9 (0.6) | 4 (0.4) | 5 (0.8) |  |
| brain | 3 (0.2) | 1 (0.1) | 2 (0.4) |  |
| breast | 152 (16.4) | 152 (27.3) | 0 (0.0) |  |
| cervix (cervical) | 78 (9.5) | 78 (15.7) | 0 (0.0) |  |
| colon | 73 (4.7) | 33 (4.1) | 40 (5.7) |  |
| esophagus (esophageal) | 3 (0.3) | 1 (0.3) | 2 (0.3) |  |
| kidney | 18 (1.5) | 8 (1.6) | 10 (1.4) |  |
| larynx/ windpipe | 8 (0.6) | 0 (0.0) | 8 (1.6) |  |
| leukemia | 10 (1.0) | 3 (0.5) | 7 (1.7) |  |
| liver | 1 (0.0) | 0 (0.0) | 1 (0.1) |  |
| lung | 27 (2.6) | 10 (2.1) | 17 (3.5) |  |
| lymphoma/hodgkin's disease | 21 (3.4) | 9 (2.7) | 12 (4.5) |  |
| melanoma | 72 (9.1) | 35 (9.1) | 37 (9.0) |  |
| mouth/tongue/lip | 9 (0.9) | 2 (0.7) | 7 (1.3) |  |
| other | 47 (4.3) | 26 (4.2) | 21 (4.5) |  |
| ovary (ovarian) | 24 (2.8) | 24 (4.6) | 0 (0.0) |  |
| prostate | 158 (9.8) | 0 (0.0) | 158 (24.7) |  |
| rectum (rectal) | 6 (0.5) | 2 (0.3) | 4 (0.8) |  |
| skin (non-melanoma) | 196 (21.8) | 77 (15.7) | 119 (31.1) |  |
| soft tissue (muscle or fat) | 3 (0.3) | 1 (0.2) | 2 (0.5) |  |
| stomach | 7 (0.5) | 3 (0.5) | 4 (0.4) |  |
| testis (testicular) | 7 (1.0) | 0 (0.0) | 7 (2.5) |  |
| thyroid | 16 (2.2) | 12 (2.9) | 4 (1.1) |  |
| uterus (uterine) | 49 (4.1) | 49 (6.8) | 0 (0.0) |  |
| Death (cancer) n (weighted %) | |  |  | p<0.05 |
| no | 14204 (96.0) | 7110 (96.7) | 7094 (95.4) |  |
| yes | 731 (4.0) | 271 (3.3) | 460 (4.6) |  |
| Death (CVD) n (weighted %) | |  |  | 0.357 |
| no | 14031 (95.7) | 7001 (95.8) | 7030 (95.5) |  |
| yes | 904 (4.3) | 380 (4.2) | 524 (4.5) |  |
| NLR, mean (SD) | 2.29 (1.16) | 2.34 (1.17) | 2.23 (1.14) | 0.298 |
| Arm circumference Ac, cm mean (SD) | 31.27 (4.16) | 30.03 (4.14) | 32.48 (3.64) | p<0.05 |
| Triceps skinfold TSF, mm mean (SD) | 17.66 (8.08) | 22.32 (7.29) | 13.12 (5.94) | p<0.05 |
| Subscapular skinfold SSF, mm mean (SD) | 19.22 (7.62) | 20.17 (8.04) | 18.28 (7.12) | p<0.05 |
| Thigh circumference TC, cm mean (SD) | 51.34 (5.91) | 50.83 (6.28) | 51.84 (5.41) | p<0.05 |
| Maximal calf circumference CC, cm mean (SD) | 37.33 (3.76) | 36.72 (3.85) | 37.92 (3.50) | p<0.05 |
| Weight, kg mean (SD) | 74.31 (16.54) | 67.98 (14.30) | 80.49 (15.31) | p<0.05 |
| BMI, kg/m^2^ mean (SD) | 26.28 (4.72) | 26.20 (5.09) | 26.36 (4.28) | p<0.05 |
| Waist circumference, cm mean (SD) | 92.37 (13.33) | 89.63 (12.90) | 95.05 (12.63) | p<0.05 |
| SWR mean (SD) | 0.20 (0.07) | 0.22 (0.07) | 0.19 (0.06) | p<0.05 |
| AWR mean (SD) | 0.34 (0.03) | 0.34 (0.03) | 0.34 (0.03) | p<0.05 |
| CWR mean (SD) | 0.41 (0.05) | 0.41 (0.05) | 0.40 (0.04) | p<0.05 |
| WWR mean (SD) | 0.80 (0.10) | 0.76 (0.08) | 0.84 (0.08) | p<0.05 |
| WHtR mean (SD) | 0.55 (0.08) | 0.56 (0.08) | 0.55 (0.07) | 0.902 |
| STR mean (SD) | 1.21 (0.49) | 0.92 (0.27) | 1.49 (0.49) | p<0.05 |
| TSFWR mean (SD) | 0.19 (0.08) | 0.25 (0.07) | 0.14 (0.05) | p<0.05 |
| TCWR mean (SD) | 0.56 (0.07) | 0.57 (0.07) | 0.55 (0.06) | p<0.05 |
| TSFCCR mean (SD) | 0.47 (0.20) | 0.60 (0.17) | 0.34 (0.14) | p<0.05 |
| TSFTCR mean (SD) | 0.34 (0.14) | 0.43 (0.12) | 0.25 (0.10) | p<0.05 |

Note: SWR: subscapular skinfold-to-waist circumference ratio; AWR: arm circumference-to-waist circumference ratio; CWR: maximal calf circumference-to-waist circumference ratio; TSFWR: triceps skinfold-to-waist circumference ratio; TCWR: thigh circumference-to-waist circumference ratio; WWR: weight-to-waist circumference ratio; WHtR: waist circumference-to-height ratio; STR: subscapular-to-triceps skinfold thickness ratio; TSFCCR: triceps skinfold-to-maximal calf circumference ratio; TSFTCR: triceps skinfold-to-thigh circumference ratio.

Table S2 Sample size and characteristics, NHANES 1999 to 2006 with DXA data.

| 1999-2006 | Total (13506) | Female (6278) | Male (7228) | P value |
| --- | --- | --- | --- | --- |
| Sex n (weighted %) | |  |  |  |
| Female | 6278 (48.2) | 6278 (100.0) | 0 | p<0.05 |
| Male | 7228 (51.8) | 0 | 7228 (100.0) |  |
| Age, year mean (SD) | 45.26 (16.10) | 46.12 (16.44) | 44.52 (15.71) | p<0.05 |
| Age n (weighted %) | |  |  |  |
| <45 year | 6997 (55.0) | 3141 (52.8) | 3856 (57.1) | p<0.05 |
| 45-65 year | 3634 (33.5) | 1727 (34.0) | 1907 (33.1) |  |
| ≥65 year | 2875 (11.4) | 1410 (13.1) | 1465 (9.8) |  |
| BMI n (weighted %) | |  |  |  |
| <20 kg/m^2^ | 4805 (36.1) | 2361 (40.8) | 2444 (31.6) | p<0.05 |
| 20-25 kg/m^2^ | 1000 (7.2) | 578 (10.1) | 422 (4.5) |  |
| 25-30 kg/m^2^ | 5119 (37.3) | 2049 (30.1) | 3070 (43.9) |  |
| ≥30 kg/m^2^ | 2582 (19.5) | 1290 (19.0) | 1292 (20.0) |  |
| Ethnic n (weighted %) | |  |  |  |
| Mexican American | 3240 (7.5) | 1475 (6.3) | 1765 (8.7) | p<0.05 |
| Non-Hispanic Black | 2522 (9.0) | 1107 (8.8) | 1415 (9.2) |  |
| Non-Hispanic White | 6657 (73.5) | 3153 (74.0) | 3504 (73.1) |  |
| Other Hispanic | 571 (4.7) | 293 (5.1) | 278 (4.3) |  |
| Other Race - Including Multi-Racial | 516 (5.2) | 250 (5.8) | 266 (4.7) |  |
| CVD n (weighted %) | |  |  |  |
| no | 11837 (91.9) | 5561 (92.1) | 6276 (91.7) | 0.552 |
| yes | 1669 (8.1) | 717 (7.9) | 952 (8.3) |  |
| Tumor n (weighted %) | |  |  | p<0.05 |
| no | 10878 (93.0) | 5031 (91.1) | 5847 (94.8) |  |
| yes | 951 (7.0) | 494 (8.8) | 457 (5.2) |  |
| Tumor n (weighted %) | |  |  |  |
| bladder | 18 (1.1) | 3 (0.2) | 15 (2.5) | p<0.05 |
| blood | 2 (0.3) | 0 | 2 (0.7) |  |
| bone | 9 (0.4) | 4 (0.3) | 5 (0.6) |  |
| brain | 2 (0.1) | 1 (0.1) | 1 (0.3) |  |
| breast | 127 (15.3) | 127 (24.9) | 0 |  |
| cervix (cervical) | 68 (11.6) | 68 (19.0) | 0 |  |
| colon | 63 (3.8) | 27 (2.9) | 36 (5.2) |  |
| esophagus (esophageal) | 3 (0.3) | 1 (0.3) | 2 (0.2) |  |
| kidney | 16 (1.3) | 7 (1.5) | 9 (0.9) |  |
| larynx/ windpipe | 8 (0.5) | 0 | 8 (1.3) |  |
| leukemia | 9 (0.7) | 2 (0.3) | 7 (1.3) |  |
| liver | 0 | 0 | 0 |  |
| lung | 23 (2.0) | 9 (1.4) | 14 (2.8) |  |
| lymphoma/hodgkin's disease | 19 (4.4) | 8 (2.9) | 11 (6.7) |  |
| melanoma | 59 (8.3) | 32 (9.8) | 27 (5.9) |  |
| mouth/tongue/lip | 8 (1.1) | 2 (1.1) | 6 (1.2) |  |
| other | 126 (12.7) | 62 (9.8) | 64 (16.3) |  |
| ovary (ovarian) | 23 (2.4) | 23 (3.9) | 0 |  |
| prostate | 129 (6.8) | 0 | 129 (17.6) |  |
| rectum (rectal) | 6 (0.6) | 2 (0.2) | 4 (1.2) |  |
| skin (non-melanoma) | 161 (19.4) | 60 (11.5) | 101 (32.0) |  |
| soft tissue (muscle or fat) | 3 (0.2) | 1 (0.2) | 2 (0.4) |  |
| stomach | 7 (0.8) | 3 (0.9) | 4 (0.6) |  |
| testis (testicular) | 6 (0.6) | 0 | 6 (1.6) |  |
| thyroid | 16 (2.5) | 12 (3.5) | 4 (0.9) |  |
| uterus (uterine) | 40 (3.4) | 40 (5.5) | 0 |  |
| Death (cancer) n (weighted %) | |  |  |  |
| no | 12845 (96.7) | 6034 (97.2) | 6811 (96.3) | 0.008 |
| yes | 661 (3.3) | 244 (2.8) | 417 (3.7) |  |
| Death (CVD) n (weighted %) | |  |  |  |
| no | 12723 (96.9) | 5951 (96.9) | 6772 (96.8) | 0.657 |
| yes | 783 (3.1) | 327 (3.1) | 456 (3.2) |  |
| NLR, mean (SD) | 2.18 (1.08) | 2.14 (1.05) | 2.22 (1.11) | 0.211 |
| Weight, kg mean (SD) | 74.58 (16.80) | 67.67 (14.56) | 80.57 (15.41) | p<0.05 |
| BMI, kg/m^2^ mean (SD) | 26.23 (4.75) | 26.09 (5.14) | 26.36 (4.30) | p<0.05 |
| Left Leg Fat, g mean (SD) | 4181.92 (1675.77) | 4982.13 (1723.48) | 3486.88 (1301.78) | p<0.05 |
| Right Leg Fat, g mean (SD) | 4296.62 (1722.34) | 5122.41 (1771.56) | 3579.37 (1337.00) | p<0.05 |
| Trunk Fat, g mean (SD) | 11714.34 (5198.20) | 12348.05 (5329.94) | 11163.92 (5059.67) | p<0.05 |
| LTrR, mean (SD) | 0.79 (0.27) | 0.89 (0.29) | 0.69 (0.20) | p<0.05 |

LTrR: leg fat-to-trunk fat ratio.

Table S3 Sample size and characteristics, NHANES 2011 to 2018 with DXA data.

| **2011-2018** | **Total (10644)** | **Female (5234)** | **Male (5410)** | **P value** |
| --- | --- | --- | --- | --- |
| Sex n (weighted %) | |  |  |  |
| Female | 5234 (48.3) | 5234 (100.0) | 0 | p<0.05 |
| Male | 5410 (51.7) | 0 | 5410 (100.0) |  |
| Age, year mean (SD) | 37.46 (12.24) | 37.88 (12.16) | 37.05 (12.31) | 0.061 |
| Age n (weighted %) | |  |  |  |
| <45 year | 7084 (64.7) | 3429 (63.6) | 3655 (65.6) | 0.103 |
| 45-65 year | 3560 (35.3) | 1805 (36.4) | 1755 (34.4) |  |
| ≥65 year |  |  |  |  |
| BMI n (weighted %) | |  |  |  |
| <20 kg/m^2^ | 2964 (27.3) | 1453 (29.7) | 1511 (25.1) | p<0.05 |
| 20-25 kg/m^2^ | 637 (5.4) | 395 (6.9) | 242 (4.1) |  |
| 25-30 kg/m^2^ | 3362 (32.8) | 1395 (27.8) | 1967 (37.5) |  |
| ≥30 kg/m^2^ | 3681 (34.5) | 1991 (35.6) | 1690 (33.4) |  |
| Ethnic n (weighted %) | |  |  |  |
| Mexican American | 1673 (11.0) | 830 (10.2) | 843 (11.6) | 0.099 |
| Non-Hispanic Black | 2161 (10.8) | 1067 (11.1) | 1094 (10.5) |  |
| Non-Hispanic White | 3620 (60.9) | 1765 (61.1) | 1855 (60.7) |  |
| Other Hispanic | 1127 (7.5) | 605 (7.6) | 522 (7.4) |  |
| Other Race - Including Multi-Racial | 2063 (9.8) | 967 (9.9) | 1096 (9.7) |  |
| CVD n (weighted %) | |  |  |  |
| no | 10280 (97.1) | 5057 (97.2) | 5223 (97.0) | 0.516 |
| yes | 364 (2.9) | 177 (2.8) | 187 (3.0) |  |
| Tumor n (weighted %) | |  |  | p<0.05 |
| no | 9448 (95.5) | 4616 (94.6) | 4832 (96.4) |  |
| yes | 334 (4.4) | 218 (5.3) | 116 (3.6) |  |
| Tumor type n (weighted %) | |  |  | p<0.05 |
| bladder | 1 (0.2) | 1 (0.3) | 0 | p<0.05 |
| blood | 2 (0.4) | 1 (0.4) | 1 (0.2) |  |
| bone | 1 (0.4) | 0 | 1 (1.1) |  |
| brain | 4 (0.7) | 3 (0.6) | 1 (0.8) |  |
| breast | 45 (9.8) | 45 (16.8) | 0 |  |
| cervix (cervical) | 46 (14.1) | 46 (24.2) | 0 |  |
| colon | 14 (2.6) | 10 (3.5) | 4 (1.3) |  |
| esophagus (esophageal) | 2 (0.3) | 0 | 2 (0.7) |  |
| kidney | 6 (1.5) | 2 (0.5) | 4 (2.9) |  |
| larynx/ windpipe | 1 (0.3) | 0 | 1 (0.7) |  |
| leukemia | 8 (2.4) | 2 (0.8) | 6 (4.7) |  |
| liver | 2 (0.2) | 0 | 2 (0.6) |  |
| lung | 5 (0.9) | 3 (0.7) | 2 (1.1) |  |
| lymphoma/hodgkin's disease | 8 (1.8) | 3 (1.1) | 5 (2.8) |  |
| melanoma | 22 (9.9) | 11 (8.3) | 11 (12.2) |  |
| mouth/tongue/lip | 2 (0.6) | 1 (0.2) | 1 (1.2) |  |
| other | 53 (18.3) | 31 (14.3) | 22 (23.7) |  |
| ovary (ovarian) | 12 (3.3) | 12 (5.6) | 0 |  |
| prostate | 1 (0.1) | 1 (0.2) | 0 |  |
| rectum (rectal) | 14 (2.7) | 0 | 14 (6.5) |  |
| skin (non-melanoma) | 1 (0.1) | 0 | 1 (0.2) |  |
| soft tissue (muscle or fat) | 41 (19.0) | 14 (10.9) | 27 (30.5) |  |
| stomach | 2 (0.7) | 1 (1.0) | 1 (0.3) |  |
| testis (testicular) | 7 (2.6) | 0 | 7 (6.2) |  |
| thyroid | 17 (4.7) | 14 (6.4) | 3 (2.4) |  |
| uterus (uterine) | 17 (2.5) | 17 (4.2) | 0 |  |
| Death (cancer) n (weighted %) | |  |  |  |
| no | 10608 (99.6) | 5214 (99.6) | 5394 (99.6) | 0.886 |
| yes | 36 (0.4) | 20 (0.4) | 16 (0.4) |  |
| Death (CVD) n (weighted %) | |  |  |  |
| no | 10614 (99.8) | 5221 (99.8) | 5393 (99.7) | 0.286 |
| yes | 30 (0.2) | 13 (0.2) | 17 (0.3) |  |
| NLR, mean (SD) | 2.01 (0.98) | 2.02 (0.94) | 2.01 (1.01) | 0.468 |
| Weight, kg mean (SD) | 80.19 (20.27) | 75.00 (19.98) | 85.22 (18.91) | p<0.05 |
| BMI, kg/m^2^ mean (SD) | 28.46 (6.52) | 28.86 (7.25) | 28.07 (5.76) | 0.077 |
| Left Leg Fat, g mean (SD) | 4696.73 (2078.17) | 5532.79 (2146.42) | 3887.86 (1672.20) | p<0.05 |
| Right Leg Fat, g mean (SD) | 4813.68 (2111.32) | 5673.33 (2179.56) | 3981.99 (1691.43) | p<0.05 |
| Trunk Fat, g mean (SD) | 12781.48 (6238.65) | 13867.62 (6538.87) | 11730.67 (5827.05) | p<0.05 |
| LTrR, mean (SD) | 0.80 (0.25) | 0.88 (0.26) | 0.73 (0.20) | p<0.05 |

LTrR: leg fat-to-trunk fat ratio.

Table S4 cut-off value of ten fat distribution indicators, NHANES 1999 to 2006, adjusted by age, ethnicity, NLR.

| **Indicator** | **Cut-off** |
| --- | --- |
| SWR | 0.18 |
| AWR | 0.35 |
| CWR | 0.40 |
| WWR | 0.75 |
| TSFWR | 0.25 |
| TCWR | 0.55 |
| WHtR | 0.50 |
| STR | 1.00 |
| TSFCCR | 0.20 |
| male | 0.20, 0.60 |
| female | 0.50, 0.90 |
| TSFTCR |  |
| male | 0.15, 0.45 |
| female | 0.43, 0.77 |
| TSF |  |
| male | 10.5 |
| female | 20.3 |

Note: SWR: subscapular skinfold-to-waist circumference ratio; AWR: arm circumference-to-waist circumference ratio; CWR: maximal calf circumference-to-waist circumference ratio; TSFWR: triceps skinfold-to-waist circumference ratio; TCWR: thigh circumference-to-waist circumference ratio; WWR: weight-to-waist circumference ratio; WHtR: waist circumference-to-height ratio; STR: subscapular-to-triceps skinfold thickness ratio; TSFCCR: triceps skinfold-to-maximal calf circumference ratio; TSFTCR: triceps skinfold-to-thigh circumference ratio.

Table S5 the association of ten indictors and all-cause mortality, NHANES 1999 to 2006, adjusted by age, ethnicity, NLR.

| **As continues** | **crude HR (95%CI)** | **P value** | **adjusted HR (95%CI)** | **P value** |
| --- | --- | --- | --- | --- |
| SWR | 0.028(0.014,0.055) | p<0.05 | 0.063(0.033,0.123) | p<0.05 |
| AWR | <0.001 (<0.001, <0.001) | p<0.05 | <0.001(<0.001,0.001) | p<0.05 |
| CWR | <0.001 (<0.001, <0.001) | p<0.05 | 0.001(<0.001,0.004) | p<0.05 |
| WWR | 0.002(0.001,0.003) | p<0.05 | 0.323(0.196,0.533) | p<0.05 |
| TSFWR | 0.098(0.056,0.171) | p<0.05 | 0.054(0.029,0.101) | p<0.05 |
| TCWR | <0.001 (<0.001, <0.001) | p<0.05 | 0.005(0.002,0.014) | p<0.05 |
| WHtR | 494.609(318.375,768.397) | p<0.05 | 2.400(1.410,4.084) | 0.001 |
| STR | 0.973(0.893,1.059) | 0.526 | 1.159(1.068,1.258) | p<0.05 |
| TSFCCR | 1.054(0.844,1.316) | 0.646 | 0.454(0.359,0.574) | p<0.05 |
| TSFTCR | 1.441(1.071,1.939) | 0.016 | 0.309(0.224,0.428) | p<0.05 |

Note: SWR: subscapular skinfold-to-waist circumference ratio; AWR: arm circumference-to-waist circumference ratio; CWR: maximal calf circumference-to-waist circumference ratio; TSFWR: triceps skinfold-to-waist circumference ratio; TCWR: thigh circumference-to-waist circumference ratio; WWR: weight-to-waist circumference ratio; WHtR: waist circumference-to-height ratio; STR: subscapular-to-triceps skinfold thickness ratio; TSFCCR: triceps skinfold-to-maximal calf circumference ratio; TSFTCR: triceps skinfold-to-thigh circumference ratio.

Table S6 sensitive analysis: the association of ten indictors (cut-off) and all-cause mortality, NHANES 1999 to 2006, adjusted by age, ethnicity, NLR.

|  | **Participants with CVD** | | **Participants with cancer** | | **Participants without CVD or cancer** | | **Old participants ≥(65 year)** | | **Young participants (<65 year)** | |
| --- | --- | --- | --- | --- | --- | --- | --- | --- | --- | --- |
|  | **aHR (95%CI)** | **P value** | **aHR (95%CI)** | **P value** | **aHR (95%CI)** | **P value** | **aHR (95%CI)** | **P value** | **aHR (95%CI)** | **P value** |
| **SWR** |  |  |  |  |  |  |  |  |  |  |
| High | ref. |  | ref. |  | ref. |  | ref. |  | ref. |  |
| Low | 1.248(1.072,1.453) | p<0.05 | 1.524(1.290,1.802) | p<0.05 | 1.273(1.132,1.431) | p<0.05 | 1.244(1.129,1.370) | p<0.05 | 1.250(1.063,1.470) | p<0.05 |
| **AWR** |  |  |  |  |  |  |  |  |  |  |
| High | ref. |  | ref. |  | ref. |  | ref. |  | ref. |  |
| Low | **1.337(1.098,1.629)** | p<0.05 | **1.642(1.279,2.109)** | p<0.05 | 1.459(1.268,1.679) | p<0.05 | **1.375(1.188,1.592)** | p<0.05 | 1.612(1.364,1.905) | p<0.05 |
| **CWR** |  |  |  |  |  |  |  |  |  |  |
| High | ref. |  | ref. |  | ref. |  | ref. |  | ref. |  |
| Low | 1.281(1.090,1.505) | p<0.05 | 1.433(1.227,1.673) | p<0.05 | **1.552(1.367,1.762)** | p<0.05 | 1.317(1.176,1.475) | p<0.05 | **1.968(1.628,2.380)** | p<0.05 |
| **WWR** |  |  |  |  |  |  |  |  |  |  |
| High | ref. |  | ref. |  | ref. |  | ref. |  | ref. |  |
| Low | 1.205(1.041,1.396) | p<0.05 | 1.238(1.024,1.498) | p<0.05 | 1.130(1.010,1.264) | p<0.05 | 1.035(0.944,1.135) | **0.467** | 1.241(1.058,1.454) | p<0.05 |
| **TSFWR** |  |  |  |  |  |  |  |  |  |  |
| High | ref. |  | ref. |  | ref. |  | ref. |  | ref. |  |
| Low | 1.295(1.092,1.536) | p<0.05 | **1.641(1.260,2.138)** | p<0.05 | **1.625(1.433,1.844)** | p<0.05 | **1.506(1.330,1.705)** | p<0.05 | 1.707(1.395,2.088) | p<0.05 |
| **TCWR** |  |  |  |  |  |  |  |  |  |  |
| High | ref. |  | ref. |  | ref. |  | ref. |  | ref. |  |
| Low | **1.307(1.079,1.583)** | p<0.05 | 1.55(1.234,1.947) | p<0.05 | 1.486(1.325,1.667) | p<0.05 | 1.356(1.208,1.521) | p<0.05 | **1.859(1.589,2.175)** | p<0.05 |
| **WHtR** |  |  |  |  |  |  |  |  |  |  |
| High | ref. |  | ref. |  | ref. |  | ref. |  | ref. |  |
| Low | 1.108(0.876,1.403) | **0.393** | 1.124(0.892,1.415) | **0.322** | 1.044(0.902,1.208) | **0.562** | 1.126(0.991,1.279) | **0.069** | 0.912(0.758,1.098) | **0.331** |
| **STR** |  |  |  |  |  |  |  |  |  |  |
| High | ref. |  | ref. |  | ref. |  | ref. |  | ref. |  |
| Low | 1.083(0.934,1.255) | **0.290** | 1.005(0.872,1.159) | **0.945** | 0.807(0.723,0.900) | p<0.05 | 0.855(0.782,0.936) | p<0.05 | 0.817(0.692,0.965) | p<0.05 |
| **TSFCCR** |  |  |  |  |  |  |  |  |  |  |
| Low | 1.350(1.096,1.662) | p<0.05 | 1.156(0.929,1.438) | 0.194 | 1.297(1.132,1.486) | p<0.05 | 1.153(1.024,1.298) | p<0.05 | 1.310(1.096,1.565) | p<0.05 |
| Normal | ref. |  | ref. |  | ref. |  | ref. |  | ref. |  |
| High | 1.071(0.771,1.490) | 0.682 | 1.222(0.746,2.004) | 0.426 | 1.564(1.194,2.050) | p<0.05 | 1.283(1.038,1.586) | p<0.05 | 1.702(1.224,2.366) | p<0.05 |
| **TSFTCR** |  |  |  |  |  |  |  |  |  |  |
| Low | 1.217(0.996,1.487) | 0.055 | 1.198(0.973,1.475) | 0.089 | 1.120(1.015,1.236) | p<0.05 | 1.079(0.970,1.201) | 0.160 | 1.085(0.923,1.275) | 0.321 |
| Normal | ref. |  | ref. |  | ref. |  | ref. |  | ref. |  |
| High | 1.166(0.768,1.771) | 0.470 | 1.126(0.57,2.222) | 0.733 | 1.725(1.168,2.546) | p<0.05 | 1.367(0.972,1.922) | 0.072 | 1.878(1.152,3.062) | p<0.05 |
| **BMI** |  |  |  |  |  |  |  |  |  |  |
| Low | 1.655(1.132,2.418) | p<0.05 | 1.683(1.028,2.756) | p<0.05 | 1.662(1.323,2.087) | p<0.05 | 1.508(1.193,1.905) | p<0.05 | 1.796(1.274,2.530) | p<0.05 |
| Normal | ref. |  | ref. |  | ref. |  | ref. |  | ref. |  |
| High | **1.043(0.883,1.232)** | **0.624** | **0.940(0.772,1.146)** | **0.542** | **1.170(1,100.368)** | **0.050** | **1.059(0.944,1.188)** | **0.326** | 1.299(1.065,1.584) | p<0.05 |
| **TSF** |  |  |  |  |  |  |  |  |  |  |
| High | ref. |  | ref. |  | ref. |  | ref. |  | ref. |  |
| Low | 1.104(0.944,1.291) | 0.215 | 1.300(1.08,1.564) | p<0.05 | 1.175(1.066,1.295) | p<0.05 | 1.101(1.006,1.205) | p<0.05 | 1.181(0.994,1.403) | 0.059 |

Note: SWR: subscapular skinfold-to-waist circumference ratio; AWR: arm circumference-to-waist circumference ratio; CWR: maximal calf circumference-to-waist circumference ratio; TSFWR: triceps skinfold-to-waist circumference ratio; TCWR: thigh circumference-to-waist circumference ratio; WWR: weight-to-waist circumference ratio; WHtR: waist circumference-to-height ratio; STR: subscapular-to-triceps skinfold thickness ratio; TSFCCR: triceps skinfold-to-maximal calf circumference ratio; TSFTCR: triceps skinfold-to-thigh circumference ratio.

Table S7 Logistic regression analysis of the association between CVD prevalence and ten indictors (cut-off) . NHANES 1999 to 2006, adjusted by age, ethnicity, NLR.

|  | **CVD Prevalence** | | **Cancer Prevalence** | |
| --- | --- | --- | --- | --- |
|  | **aOR(95%CI)** | **P value** | **aOR(95%CI)** | **P value** |
| SWR≥0.18 | ref. |  | ref. |  |
| SWR<0.18 | 0.813(0.715,0.924) | p<0.05 | 0.926(0.808,1.059) | 0.261 |
| AWR≥0.35 | ref. |  | ref. |  |
| AWR<0.35 | 1.226(1.038,1.453) | p<0.05 | 1.155(0.969,1.382) | 0.110 |
| CWR≥0.4 | ref. |  | ref. |  |
| CWR<0.4 | 1.656(1.434,1.915) | p<0.05 | 1.027(0.886,1.190) | 0.726 |
| WWR≥0.75 | ref. |  | ref. |  |
| WWR<0.75 | 0.831(0.728,0.949) | p<0.05 | 0.839(0.727,0.967) | p<0.05 |
| WHtR≥0.5 | ref. |  | ref. |  |
| WHtR<0.5 | 0.644(0.527,0.782) | p<0.05 | 1.168(0.974,1.395) | 0.090 |
| STR≥1 | ref. |  | ref. |  |
| STR<1 | 0.742(0.652,0.843) | p<0.05 | 1.007(0.881,1.151) | 0.917 |
| TSFWR≥0.25 | ref. |  | ref. |  |
| TSFWR<0.25 | 1.200(1.029,1.402) | p<0.05 | 0.846(0.725,0.989) | p<0.05 |
| TCWR≥0.55 | ref. |  | ref. |  |
| TCWR<0.55 | 1.802(1.535,2.121) | p<0.05 | 0.999(0.850,1.175) | 0.989 |
| BMI<20 | 1.112(0.841,1.455) | 0.446 | 1.074(0.805,1.413) | 0.619 |
| BMI=20~30 | ref. |  | ref. |  |
| BMI≥30 | 1.491(1.292,1.719) | p<0.05 | 1.026(0.870,1.206) | 0.757 |
| low TSFCCR | 0.810(0.683,0.958) | p<0.05 | 1.051(0.885,1.244) | 0.566 |
| normal TSFCCR | ref. |  | ref. |  |
| high TSFCCR | 1.310(1.005,1.691) | p<0.05 | 1.126(0.830,1.503) | 0.431 |
| low TSFTCR | 0.781(0.675,0.902) | p<0.05 | 0.977(0.840,1.134) | 0.758 |
| normal TSFTCR | ref. |  | ref. |  |
| high TSFTCR | 1.402(0.991,1.952) | 0.050 | 1.307(0.890,1.875) | 0.158 |

Note: SWR: subscapular skinfold-to-waist circumference ratio; AWR: arm circumference-to-waist circumference ratio; CWR: maximal calf circumference-to-waist circumference ratio; TSFWR: triceps skinfold-to-waist circumference ratio; TCWR: thigh circumference-to-waist circumference ratio; WWR: weight-to-waist circumference ratio; WHtR: waist circumference-to-height ratio; STR: subscapular-to-triceps skinfold thickness ratio; TSFCCR: triceps skinfold-to-maximal calf circumference ratio; TSFTCR: triceps skinfold-to-thigh circumference ratio.

Table S8 Obesity (defined by BMI and ten indictors) population distribution, NHANES 1999 to 2006.

|  | **BMI (<20)** | **BMI (20-30)** | **BMI(≥30)** | **summary** |
| --- | --- | --- | --- | --- |
| **SWR** |  |  |  |  |
| high | 128 | 6368 | 2522 | 8890 |
| low | 961 | 4585 | 371 | 4956 |
| summary | 1089 | 10953 | 2893 | 13846 |
| **AWR** |  |  |  |  |
| high | 463 | 4482 | 852 | 5334 |
| low | 626 | 6471 | 2041 | 8512 |
| summary | 1089 | 10953 | 2893 | 13846 |
| **CWR** |  |  |  |  |
| high | 938 | 6471 | 915 | 7386 |
| low | 151 | 4482 | 1978 | 6460 |
| summary | 1089 | 10953 | 2893 | 13846 |
| **WWR** |  |  |  |  |
| high | 384 | 7300 | 2511 | 9811 |
| low | 705 | 3653 | 382 | **4035** |
| summary | 1089 | 10953 | 2893 | 13846 |
| **TSFWR** |  |  |  |  |
| high | **66** | 2537 | 1166 | 3703 |
| low | 1023 | 8416 | 1727 | 10143 |
| summary | 1089 | 10953 | 2893 | 13846 |
| **TCWR** |  |  |  |  |
| high | 854 | 6207 | 1171 | 7378 |
| low | 235 | 4746 | 1722 | 6468 |
| summary | 1089 | 10953 | 2893 | 13846 |
| **WHtR** |  |  |  |  |
| high | **59** | 7815 | 2891 | 10706 |
| low | 1030 | 3138 | **2** | 3140 |
| summary | 1089 | 10953 | 2893 | 13846 |
| **STR** |  |  |  |  |
| high | 542 | 6763 | 1807 | 8570 |
| low | 547 | 4190 | 1086 | **5276** |
| summary | 1089 | 10953 | 2893 | 13846 |
| **TSFCCR** |  |  |  |  |
| normal | 311 | 8333 | 2427 | 10760 |
| low | 776 | 2257 | 127 | 2384 |
| high | 2 | 363 | 339 | 702 |
| summary | 1089 | 10953 | 2893 | 13846 |
| **TSFTCR** |  |  |  |  |
| normal | 248 | 7322 | 2321 | 9643 |
| low | 839 | 3478 | 393 | 3871 |
| high | 2 | 153 | 179 | 332 |
| summary | 1089 | 10953 | 2893 | 13846 |

Note: the cut-off value showed in figure s2. SWR: subscapular skinfold-to-waist circumference ratio; AWR: arm circumference-to-waist circumference ratio; CWR: maximal calf circumference-to-waist circumference ratio; TSFWR: triceps skinfold-to-waist circumference ratio; TCWR: thigh circumference-to-waist circumference ratio; WWR: weight-to-waist circumference ratio; WHtR: waist circumference-to-height ratio; STR: subscapular-to-triceps skinfold thickness ratio; TSFCCR: triceps skinfold-to-maximal calf circumference ratio; TSFTCR: triceps skinfold-to-thigh circumference ratio.

Table S9 the association of LTrR and all-cause mortality in all participants, adjusted by age, ethnicity, NLR.

|  | **HR（95%CI）** | **P value** | **adjusted HR（95%CI）** | **adjusted P value** |
| --- | --- | --- | --- | --- |
| **1999-2006** |  |  |  |  |
| as continue | 0.165(0.128,0.212) | p<0.05 | 0.590(0.464,0.750) | p<0.05 |
| LTrR<0.9 | ref. |  | ref. |  |
| LTrR≥0.9 | 0.491(0.433,0.558) | p<0.05 | 0.817(0.713,0.936) | p<0.05 |
| **2011-2018** |  |  |  |  |
| as continue | 0.129(0.053,0.316) | p<0.05 | 0.202(0.064,0.643) | p<0.05 |
| LTrR<0.9 | ref. |  | ref. |  |
| LTrR≥0.9 | 0.386(0.233,0.640) | p<0.05 | 0.468(0.267,0.820) | p<0.05 |

LTrR: leg fat-to-trunk fat ratio.

Figure S1 Flow chat.





Figure S2 Association of the combination of fat distribution and bmi and all-cause mortality, NHANES 1999 to 2006. Weighted to be nationally representative, values are expressed as n (weighted %). Adjusted by age, ethnicity, NLR. SWR: subscapular skinfold-to-waist circumference ratio; AWR: arm circumference-to-waist circumference ratio; CWR: maximal calf circumference-to-waist circumference ratio; TSFWR: triceps skinfold-to-waist circumference ratio; TCWR: thigh circumference-to-waist circumference ratio; WWR: weight-to-waist circumference ratio; WHtR: waist circumference-to-height ratio; STR: subscapular-to-triceps skinfold thickness ratio; TSFCCR: triceps skinfold-to-maximal calf circumference ratio; TSFTCR: triceps skinfold-to-thigh circumference ratio.


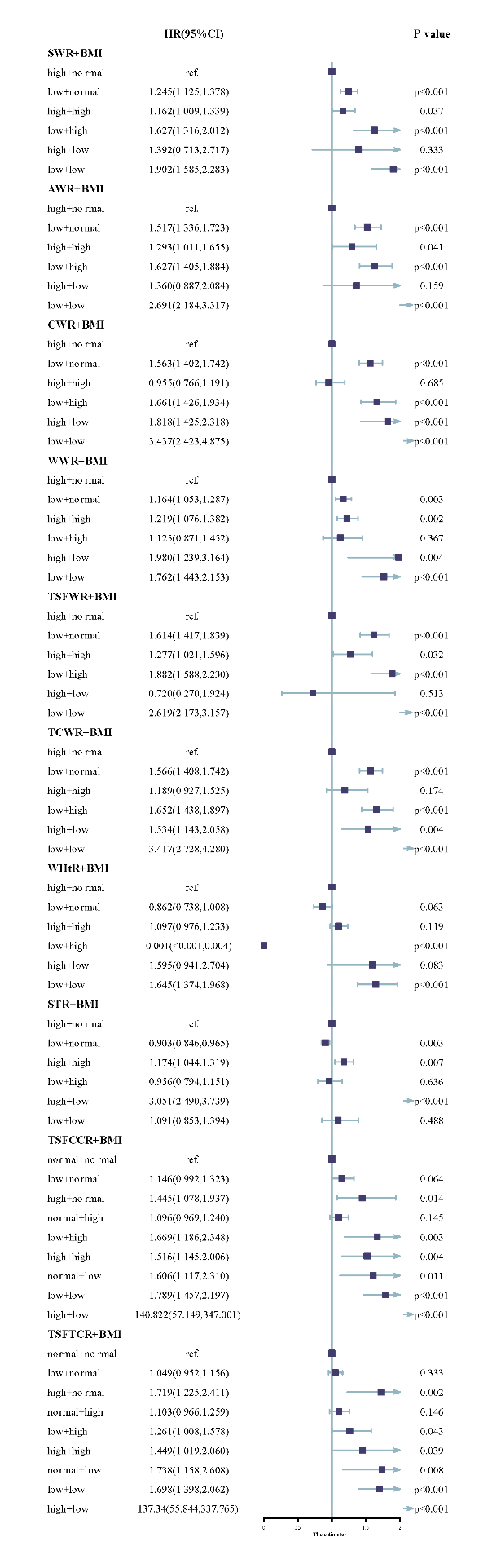


Figure S3 Restricted spline curves (weighted) examining the association of LTrR and all-cause mortality. Weighted to be nationally representative. Adjusted by age, ethnicity, NLR. LTrR = leg fat-to-trunk fat ratio.


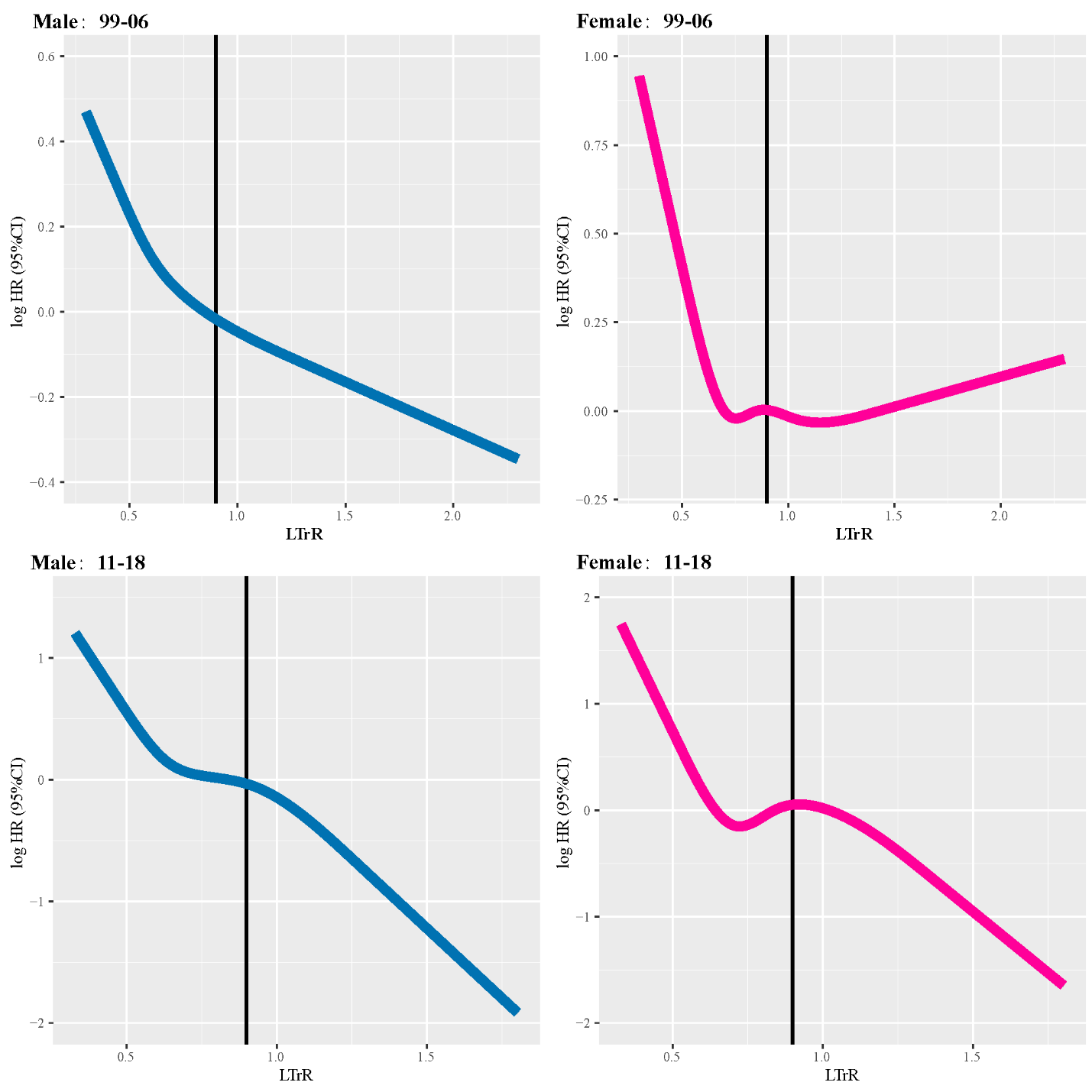

Supplement: Supplementary file 1 — Table S1 Sample size and characteristics, NHANES 1999–2006. Table S2 Sample size and characteristics, NHANES 1999–2006 with DXA data. Table S3 Sample size and characteristics, NHANES 2011–2018 with DXA data. Table S4 Cut‐off value of 10 fat distribution indicators, NHANES 1999–2006, adjusted by age, ethnicity and NLR. Table S5 The association of 10 indicators and all‐cause mortality, NHANES 1999–2006, adjusted by age, ethnicity and NLR. Table S6 Sensitive analysis: The association of 10 indicators (cut‐off) and all‐cause mortality, NHANES 1999–2006, adjusted by age, ethnicity and NLR. Table S7 Logistic regression analysis of the association between CVD prevalence and 10 indicators (cut‐off). NHANES 1999–2006, adjusted by age, ethnicity and NLR. Table S8 Obesity (defined by BMI and 10 indicators) population distribution, NHANES 1999–2006. Table S9 The association of LTrR and all‐cause mortality in all participants, adjusted by age, ethnicity and NLR. Figure S1 Flow chat. Note: SWR: subscapular skinfold‐to‐waist circumference ratio; AWR: arm circumference‐to‐waist circumference ratio; CWR: maximal calf circumference‐to‐waist circumference ratio; TSFWR: triceps skinfold‐to‐waist circumference ratio; TCWR: thigh circumference‐to‐waist circumference ratio; WWR: weight‐to‐waist circumference ratio; WHtR: waist circumference‐to‐height ratio; STR: subscapular‐to‐triceps skinfold thickness ratio; TSFCCR: triceps skinfold‐to‐maximal calf circumference ratio; TSFTCR: triceps skinfold‐to‐thigh circumference ratio. Figure S2 Association of the combination of fat distribution and BMI and all‐cause mortality, NHANES 1999–2006. Weighted to be nationally representative, values are expressed as n (weighted %). Adjusted by age, ethnicity and NLR. Note: SWR: subscapular skinfold‐to‐waist circumference ratio; AWR: arm circumference‐to‐waist circumference ratio; CWR: maximal calf circumference‐to‐waist circumference ratio; TSFWR: triceps skinfold‐to‐waist circumference ratio; TCWR: thigh [file JCSM-16-e70013-s001.docx]
